# Ventricular noncompaction in a female patient with nephropathic cystinosis: a case report

**DOI:** 10.1186/1752-1947-3-31

**Published:** 2009-01-29

**Authors:** Ibrar Ahmed, Thanh Trung Phan, Graham W Lipkin, Michael Frenneaux

**Affiliations:** 1 Department of Cardiovascular Medicine, Medical School, University of Birmingham, Edgbaston, Birmingham, B15 2TT, UK; 2 Department of Nephrology, University Hospital Birmingham, Edgbaston, Birmingham, B15 2TH, UK

## Abstract

**Introduction:**

We report an unusual and interesting case of a 24-year-old woman with nephropathic cystinosis in association with concomitant isolated noncompaction of the left ventricle. Left ventricular noncompaction usually presents with reduced exercise tolerance as a consequence of ventricular dysfunction, the result of embolus or with palpitations and syncope due to arrhythmia. There is no specific treatment directed at isolated noncompaction. Treatment is focused on the cause of presentation, with medication aimed at improving ventricular dysfunction, as well as treating and preventing thrombosis and arrhythmia.

**Case presentation:**

Our patient presented with an episode of decompensated heart failure. Trans-thoracic echocardiography demonstrated excessive trabeculation with inter-trabecular recesses in the left ventricle typical of noncompaction of the left ventricle. The patient's admission was complicated by a cardiac arrest precipitated by ventricular tachycardia for which she subsequently underwent implantation of an automatic implantable cardioverter defibrillator.

**Conclusion:**

This is, as far as we know, the first case report of the co-existence of nephropathic cystinosis and isolated noncompaction of the left ventricle. It highlights the importance of being vigilant to the diagnosis of left ventricular noncompaction.

## Introduction

Isolated noncompaction of the left ventricle (LV) is increasingly being recognized as a distinct entity with a significant associated morbidity and mortality; however, definitions are still being debated. A diagnosis can be made with the commonly available modality of echocardiography but is still often overlooked. The co-existence of noncompaction of the LV together with nephropathic cystinosis has not been described previously.

## Case presentation

A 24-year-old woman with cystinosis complicated by end-stage renal failure, for which she was receiving intermittent haemodialysis, was admitted with generalized malaise and weight loss. In May 2001, she had undergone trans-thoracic echocardiography because of increasing shortness of breath on exertion, and this had shown a moderately dilated LV with globally reduced systolic function (ejection fraction, EF = 25%), consistent with dilated cardiomyopathy.

The patient was referred for cardiology consultation following a period of increased breathlessness and a cardiac arrest precipitated by ventricular tachycardia from which she was successfully DC cardioverted. Her medication at that time included perindopril 4 mg once daily (od), carvedilol 3.125 mg twice daily (bd), prednisolone 5 mg od, levothyroxine 200 mcg od, folic acid 5 mg od, darbepoetin 80 mcg per week, mercaptamine (Cystagon) 150 mg four times per day (qds), calcium carbonate 500 mg three times daily (tds), aspirin 75 mg od and alfacalcidol 1.5 mcg od.

**Figure 1:**
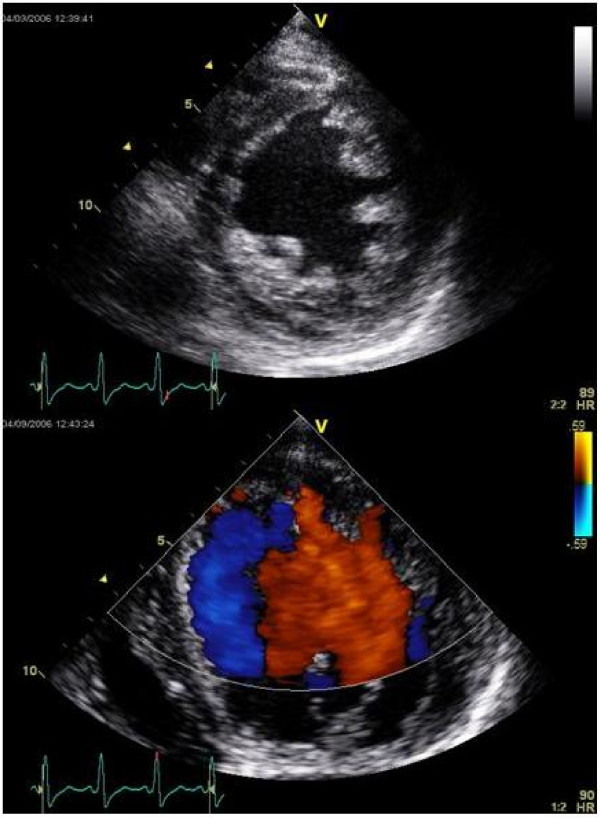
**Trans-thoracic echocardiography of our patient, demonstrating prominent trabeculation with colour-flow mapping demonstrating blood flow into the deep intertrabecular recesses in the left ventricle**.

On examination, the patient's blood pressure was 86/50 mmHg and she was in sinus rhythm at 76 bpm. She had some facial oedema, mild ankle oedema and bi-basal crackles on chest auscultation. Heart sounds examination revealed a soft apical pansystolic murmur. The remainder of the examination was unremarkable. Echocardiography revealed a dilated LV with globally impaired systolic LV function (EF Simpsons biplane = 25%). There was marked trabeculation of the LV, most prominent at the apex and lateral wall at the mid-ventricular level; six trabeculae were more than 2 mm in diameter. The noncompacted to compacted ratio was 2.2 at the thickest part of the lateral wall on the parasternal short-axis view. Multiple inter-trabecular recesses in communication with the LV cavity were demonstrated by forward and reverse flow of blood on colour flow mapping (Figure 1). These features are consistent with current diagnostic criteria for isolated ventricular noncompaction. At the time of writing, the patient had been referred for heart and kidney transplant assessment and had undergone implantation of an automatic implantable cardioverter defibrillator (AICD).

## Discussion

The first cases of cystinosis were reported in 1903 [1]. Cystinosis is an autosomal recessively inherited lysosomal storage disorder. The genetic mutation has been located on the short arm of chromosome 17 [2]. There is a failure of the normal export of cystine from the lysosome, leading to cystine accumulation in almost all cells, with variable consequences on the ability of that tissue to function normally.

Three variants have been described. These are the nephropathic, the adolescent, and the benign adult forms. Our patient had the nephropathic variant.

Skeletal muscle involvement has been widely reported. Muscle cystine concentrations approximately a thousand-fold greater than normal values are reported [3]. Cystine crystals have not been found within the myocytes themselves but in the perimysial and endomysial spaces [3]. The influence this has on the myocyte is unknown, but interestingly, there are reports of selective atrophy of type 1 muscle fibres in the skeletal muscle of patients with cystinosis [3]. Cystine concentrations in cardiac muscle have been shown to be of the same order of magnitude as those found in skeletal muscle in patients affected with cystinosis [3], and it is possible that in the absence of therapy, cystine accumulation may lead to a macroscopically hypertrophied myocardium [4] with prominent trabeculae. There is no obvious reason to believe that cardiac muscle would be affected any differently to skeletal muscle.

Isolated ventricular noncompaction is increasingly recognised as a distinct entity with an associated significant morbidity and mortality. It was the third most commonly identified primary cardiomyopathy in an epidemiological survey of Australian children, after dilated and hypertrophic cardiomyopathy [5]. However, a lack of an understanding of its pathophysiological origins, combined with a lack of consistent diagnostic criteria, has led to this condition being under-diagnosed and frequently missed. Isolated noncompaction of the ventricular myocardium may manifest itself any time from infancy to adulthood but most commonly occurs in adulthood, with men and women being affected equally frequently. Embryologically, the myocardium begins as a loose meshwork of interwoven myocardial fibres. These fibres condense and become compacted; this process begins in the epicardium and progresses towards the endocardium, and from the base toward the apex. It is commonly believed that it is the arrest of this compacting process during weeks 5–8 of embryogenesis that leads to this condition. Consistent with this is the common finding of noncompaction affecting the apex and endocardium. This leads to the commonly held belief that isolated LV noncompaction is a congenital disease; however, reports of LV noncompaction are accumulating [6]. In the majority of patients, it is the LV that is involved. Right ventricular involvement is difficult to be certain of, as prominent trabeculation may be a normal variant [7].

Isolated noncompaction may occur sporadically or be inherited. Familial adult and neonatal forms have been described. The familial adult forms of the disease are transmitted as an autosomal, dominant trait in the majority of cases [8]. Neonatal forms of isolated ventricular noncompaction can be linked to mutations affecting the X chromosome associated with Barth syndrome [9], X-linked endocardial fibroelastosis and other X-linked infantile cardiomyopathies.

Clinically, patients may present with a triad of symptoms, related to LV dysfunction, arrhythmia presenting as palpitations and syncope, or with the consequences of systemic thrombo-embolism. Others may be asymptomatic and identified incidentally. LV systolic dysfunction is one of the most commonly occurring features [7,10-13]. The concept of microvascular disease as a mechanism for ventricular dysfunction is supported by the finding of ischemic subendocardial lesions in these patients [14]. Diastolic dysfunction has been described. It is suggested that this relates to the multiple prominent trabeculations, causing restrictive filling and intrinsic abnormal ventricular relaxation [15].

Diagnosis is most commonly made by two-dimensional and colour Doppler echocardiography. Commonly used criteria include the identification of excessive (more than three), prominent (more than 2 mm diameter) trabeculae with inter-trabecular recesses that penetrate deeply into the myocardium, from which blood flows directly into and out of the ventricular cavity (which is demonstrated using colour Doppler imaging), in the absence of other structural heart disease.

More objective criteria have been validated by Jenni et al. [14]. This group found that a ratio of greater than two of the end-systolic noncompacted endocardial layer, in the parasternal short axis view, to the compacted epicardial layer of myocardium using echocardiography was diagnostic. They validated this against autopsy specimens of the same myocardium.

Areas of noncompaction are found predominantly in the apex and midventricular regions of the inferior and lateral walls, with the trabeculae most frequently coursing from the free wall to the ventricular septum [16]. Hypokinesis of affected and unaffected myocardium is not unusual, with globally reduced ventricular function.

Alternative conditions with reported prominent trabeculations include hypetrophic cardiomyopathy, endocardial fibroelastosis and metastatic disease involving the myocardium. More than three prominent trabeculations is unusual in the normal ventricle [6].

Prognosis is variable. Patients not uncommonly experience sudden death, progression of their ventricular dysfunction, arrhythmias such as ventricular tachycardia, or atrial fibrillation. Thrombo-embolic complications, secondary to atrial fibrillation or thrombus in the deep recesses, are common. Many patients may also remain asymptomatic.

Management is as for heart failure, including arrhythmia management, and more liberal use of prophylactic anticoagulation agents. Increased awareness of possible complications will allow earlier institution of correct therapies. AICD therapy for haemodynamically significant arrhythmia and cardiac transplantation should be considered.

## Conclusion

This is the first report to our knowledge of LV noncompaction in a patient with cystinosis. It highlights the importance of being vigilant to the diagnosis of LV noncompaction in such patients.

## Abbreviations

AICD: automatic implantable cardiovertor defibrillator; bd: twice daily; EF: ejection fraction; LV: left ventricle/ventricular; od: once daily; qpd: four times per day; tds: three times daily.

## Consent

Written informed consent was obtained from the patient for publication of this case report and any accompanying images. A copy of the written consent is available for review by the Editor-in-Chief of this journal.

## Competing interests

The authors declare that they have no competing interests.

## Authors' contributions

IA reviewed the literature and was a major contributor in writing the manuscript. TP reviewed the patient case history and contributed to the writing of the manuscript. GL made the diagnosis of nephropathic cystinosis and provided expert opinion on the diagnosis and management of cystinosis. MF made the diagnosis of LV noncompaction and made a large contribution to the final draft of the manuscript.
